# Supplementary material for: Dengue virus exploits the host tRNA epitranscriptome to promote viral replication
Source: bioRxiv. 2023 Nov 6:2023.11.05.565734. Preprint. [Version 1] doi: 10.1101/2023.11.05.565734 (PMC10659268; doi:10.1101/2023.11.05.565734)
Supplement: 1 [file NIHPP2023.11.05.565734V1-supplement-1.pdf]

bioRxiv preprint doi: https://doi.org/10.1101/2023.11.05.565734; this version posted November 6, 2023. The copyright holder for this preprint (which was not certified by peer review) is the author/funder, who has granted bioRxiv a license to display the preprint in perpetuity. It is made available under aCC-BY 4.0 International license.

Supporting Information for
Dengue virus exploits the host tRNA epitranscriptome to promote viral replication

Cheryl Chan, Newman Siu Kwan Sze, Yuka Suzuki, Takayuki Ohira, Tsutomu Suzuki, Thomas J. Begley, Peter C. Dedon

Contents

- Figures S1-S5
- Supporting Data Table S1: LC-MS analysis of RNA modifications; separate spreadsheet
- Supporting Data Table S2: Proteomics data for ALKBH1 knockdown and over-expression versus control; separate spreadsheet
- Supporting Data Table S3: Proteomics data for DENV infection at 8 and 24 hpi; separate spreadsheet

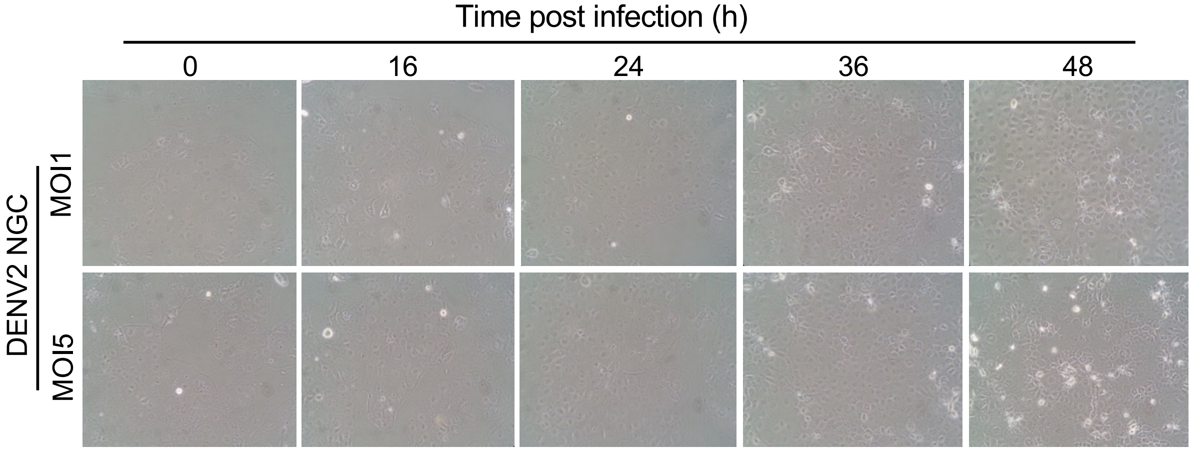

Fig. S1. Phase contrast images (10x magnification) of cells infected with DENV2 NGC at MOI 1 and 5, at indicated times post-infection.

bioRxiv preprint doi: https://doi.org/10.1101/2023.11.05.565734; this version posted November 6, 2023. The copyright holder for this preprint (which was not certified by peer review) is the author/funder, who has granted bioRxiv a license to display the preprint in perpetuity. It is made available under aCC-BY 4.0 International license.

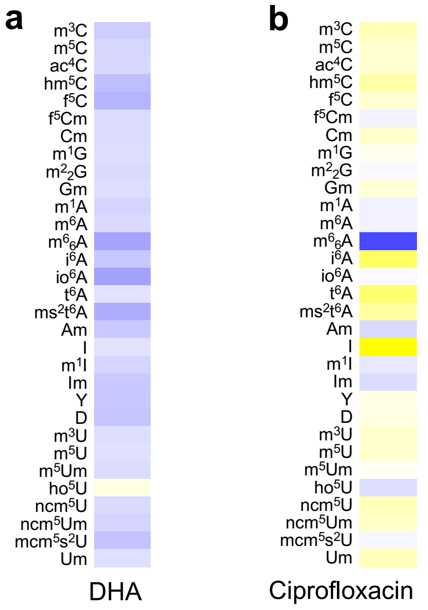

Fig. S2. tRNA modification profiles of Huh-7 cells at 24 h following treatment with (**a**) 10 μM dihydroartemisinin (DHA) and (**b**) 20 μM ciprofloxacin assessed by LC-MS/MS.

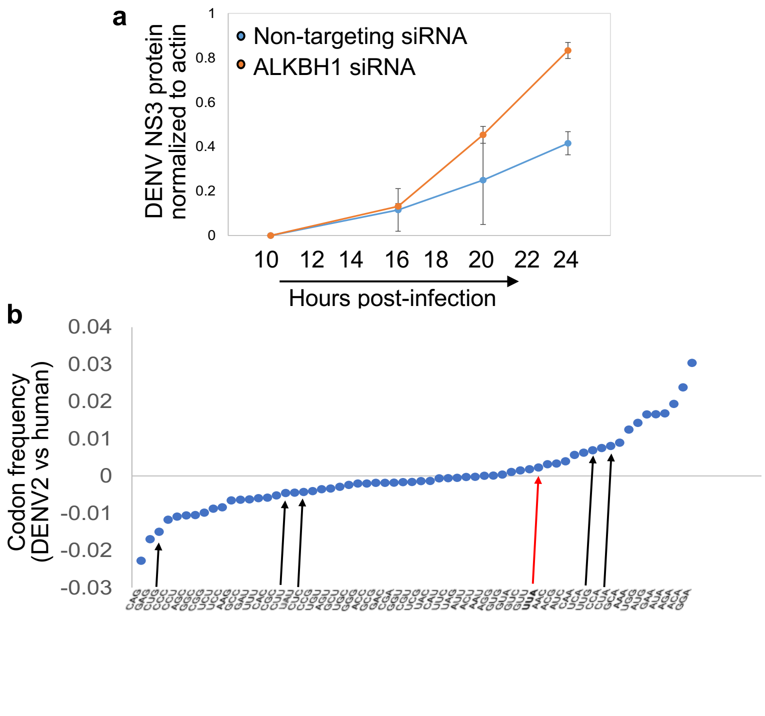

Fig. S3. (a) Densitometric quantitation of NS3 protein levels at indicated times after infection of Huh-7 cells with DENV strain EDEN2 infection. Huh-7 cells were pre-treated with control non-targeting siRNA (blue circles) and ALKBH1-specific siRNA (orange circles). **(b)** Differences in codon usage frequencies in DENV2 NGC strain versus human host genome. Leucine UUA codon (red arrow) is nearly equally represented in both human and DENV2 genomes, while other leucine codons (black arrows) are not shared equally.

bioRxiv preprint doi: https://doi.org/10.1101/2023.11.05.565734; this version posted November 6, 2023. The copyright holder for this preprint (which was not certified by peer review) is the author/funder, who has granted bioRxiv a license to display the preprint in perpetuity. It is made available under aCC-BY 4.0 International license.

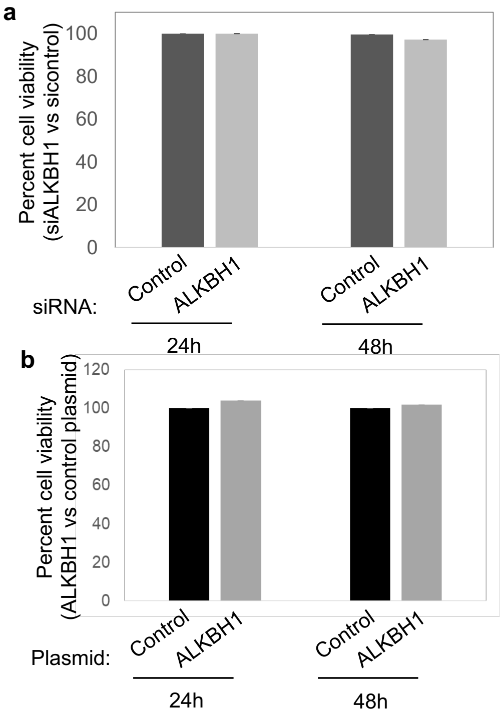

Fig. S4. Cell viability determined by MTT assay of Huh-7 cells treated with (**a**) non-targeting and ALKBH1-specific siRNA, and (**b**) control empty vector and ALKBH1 plasmid, at 24h and 48h post-transfection.

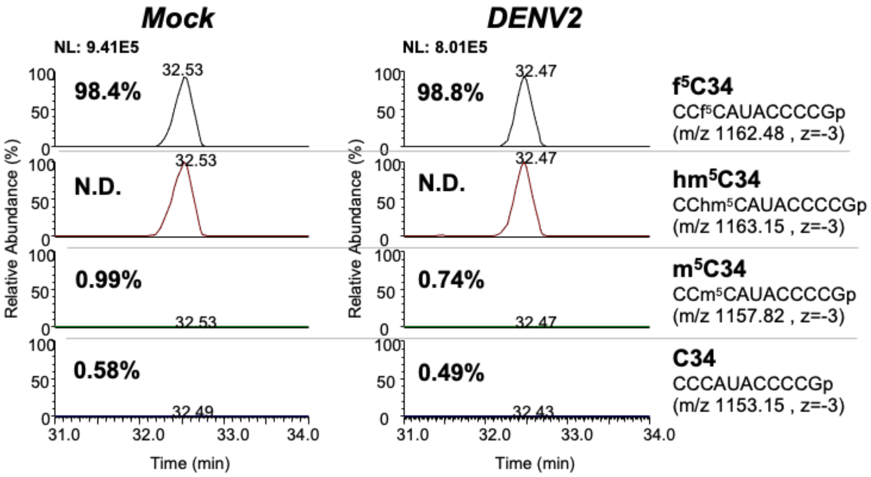

Fig. S5. Relative abundance of modifications m^5C, hm^5C and f^5C of isolated mitochondrial $tRNA^{Met}$ from mock- (left panel) and DENV2 NGC-infected Huh-7 cells (middle panel). The respective anticodon fragments harboring C34 modifications and m/z values are indicated (right panel). N.D., not detected.
